# Moniliformin Occurrence in Serbian Maize over Four Years: Understanding Weather-Dependent Variability

**DOI:** 10.3390/toxins15110634

**Published:** 2023-10-30

**Authors:** Bojana Radić, Radmila Radović, Elizabet Janić Hajnal, Anamarija Mandić, Sanja Đekić, Zorica Stojanović, Jovana Kos

**Affiliations:** 1 Institute of Food Technology in Novi Sad, University of Novi Sad, Bulevar Cara Lazara 1, 21000 Novi Sad, Serbia; radmila.radovic@fins.uns.ac.rs (R.R.); elizabet.janich@fins.uns.ac.rs (E.J.H.); anamarija.mandic@fins.uns.ac.rs (A.M.); jovana.kos@fins.uns.ac.rs (J.K.); 2 Faculty of Technology Novi Sad, University of Novi Sad, Bulevar Cara Lazara 1, 21000 Novi Sad, Serbia; zorica.stojanovic@uns.ac.rs; 3 Department of Analytical Chemistry, Faculty of Chemistry, University of Belgrade, Sudentski Trg 12-16, 11158 Belgrade, Serbia; dh012016@student.chem.bg.ac.rs

**Keywords:** emerging mycotoxin, LC-MS/MS, climate change, regional variations, annual variations

## Abstract

Moniliformin (MON) represents one of the most widespread emerging mycotoxins, whose presence in food and feed can potentially cause harmful effects on the health of both the public and animals. In order to investigate MON occurrence, a total of four hundred (n = 400) samples of unprocessed maize were sampled from different regions (Bačka, Banat, and Srem) of Serbia during a period of four years (2018–2021) and were analyzed using a validated liquid chromatography with tandem mass spectrometry (LC-MS/MS) method. The influence of regional differences and variations from year to year in terms of weather conditions on the occurrence of MON was also assessed. The obtained validation parameters indicated that the LC-MS/MS method is applicable to the determination of MON in maize samples. It can be observed from the acquired findings that all samples were contaminated with MON, while concentration levels varied between the samples, especially from different sampling years. The maize samples showed the greatest levels of MON concentration during the dry and hot climatic conditions experienced in 2021. In maize samples harvested in the period 2018–2020, the MON concentration levels detected were about two to three times lower compared to the year 2021. Additionally, a comprehensive investigation into the correlation between weather conditions and the occurrence of MON in maize grown in Serbia was undertaken by reviewing the outcomes of research undertaken in the past decade.

## 1. Introduction

Maize is one of the most important crops and occupies a significant position globally based on harvested areas and production volumes. The Republic of Serbia is one of the European leaders in the production and export of maize. Therefore, growing maize in the Republic of Serbia is a very important part of national agriculture, with a significant socio-economic impact [1]. However, fungi contamination of food and feed is one of the most important food safety concerns worldwide, as in Serbia [2,3,4,5,6,7,8].

Fungal genera members *Fusarium*, *Aspergillus*, and *Penicillium* represent the prominent fungal species that cause frequent contamination of food and feed with mycotoxins [9]. These toxigenic fungi can grow on a wide variety of crops, and after infection of crops by fungi, and under favorable conditions associated with fungal infection, they produce toxins [10]. Broad environmental conditioners, including climate change, can directly influence fungal contamination of food and feed. Moreover, alterations in temperature, particularly aligned with ongoing warming trends, along with shifts in precipitation distribution involving heightened occurrences of extreme events like floods and droughts, in addition to variations in moisture content, can collectively contribute to the achievement of overall conditions for the production of several mycotoxins [11,12]. In the last decade, climate change in Serbia has favored fungal growth, especially *Fusarium* and *Aspergillus* species, as well as mycotoxin production in maize, including regulated and non-regulated mycotoxins [2,3].

Besides widely recognized mycotoxins under regulation, there are mycotoxins defined as emerging mycotoxins that have been identified and are of concern for food and feed safety. Emerging mycotoxins are still not routinely determined, and regulatory thresholds for their presence in food and feed remain undefined. The toxicity of these mycotoxins is still under investigation, and several toxic effects are reported in scientific literature. The European Food Safety Authority (EFSA) recognized this problem and has started to publish reports to carry out a risk assessment for these toxins. However, the evidence of their occurrence is increasing, and they have become a target of the scientific community’s research due to their widespread presence in food and feed, particularly in cereals and cereal-based products [13,14,15,16].

Moniliformin (MON) is one of the most commonly occurring emerging mycotoxins produced by a number of *Fusarium* species including *F. avenaceum*, *F. verticillioides*, *F. proliferatum*, and *F. subglutinans* [13,17]. Another species of *Fusarium*, specifically *F. fujikuroi*, is known to produce several different mycotoxins such as fumonisins B_1_, B_2_, and B_3_, fusaproliferin, and beauvericin, and is also recognized to produce a considerable amount of MON [18]. Accordingly, recently published results of the co-occurrence of MON indicate that there is a connection between MON and the presence of other mycotoxins, including fumonisins, zearalenone, trichothecenes, beauvericin, and enniatins [8,19,20]. Furthermore, it has been observed that MON is not only produced by *Fusarium* species, but also one *Penicillium* species (*Penicillium melanoconidium*) is known to have the ability to produce substantial amounts of MON [21,22]. The fact that MON has become one of the most prevalent mycotoxins in maize in recent years (2016–2021) is confirmed by the most extensive available worldwide reports on the occurrence of a large number of different mycotoxins in cereals, which are conducted every year by BIOMIN [23]. According to these studies, in recent years, MON has been ranked as one of the most common mycotoxins that contaminate maize and maize products worldwide. The European Commission Directorate-General for Health and Consumers recognized the significant presence of MON in cereals, which prompted them to take action. They approached EFSA, requesting a scientific opinion on the potential risks associated with MON. Therefore, EFSA requested additional information several times (in 2010, 2012, and 2014) on the risk for public health related to the presence of MON in food and feed. Furthermore, in 2018, EFSA published a scientific assessment regarding the risks associated with the presence of MON in food and feed, which may have impacts on both human and animal health [24]. According to this scientific opinion, the data on toxicity and toxicokinetics in both experimental and farm animals are limited, indicating that MON may cause adverse health effects such as cardiotoxicity, hematotoxicity and respiratory distress. Although MON has shown the potential to cause chromosomal aberrations in vitro, but not in vivo, no carcinogenicity data have been identified. However, due to the current availability of toxicity data, it is not possible to establish human acute or chronic health-based guidance values.

Analytical methods for the determination of MON in food and feed have been mostly based on liquid chromatography with tandem mass spectrometry (LC–MS/MS). Furthermore, none of the applied analytical methods for MON have been formally validated in interlaboratory studies, and no certified reference materials for MON are commercially available. Currently, there is no available or published LC-MS/MS method for MON determination in Serbia. Nevertheless, frequent occurrences and associated health risks with MON substantially increase the demand for the development of suitable analytical methodology for the sensitive and accurate determination of this mycotoxin.

Therefore, based on all the above, the main aims of this study were (i) to develop and validate a first LC-MS/MS method in Serbia for determining MON in maize, (ii) to study the presence and levels of MON contamination in maize samples harvested in the period of four years from 2018 to 2021, (iii) to examine the influence of weather conditions on MON occurrence in the main maize growing regions of Serbia in a period of four years (2018–2021), and (iv) to provide a comparative overview of the incidence of MON in maize cultivated in Serbia in relation to weather patterns, through an analysis of studies conducted in the last decade.

## 2. Results and Discussion

The validated LC-MS/MS method was used to determine the occurrence of MON in maize depending on the production region, production year, and weather conditions observed during the maize growing season. 

### 2.1. Method Validation

**Figure 1:**
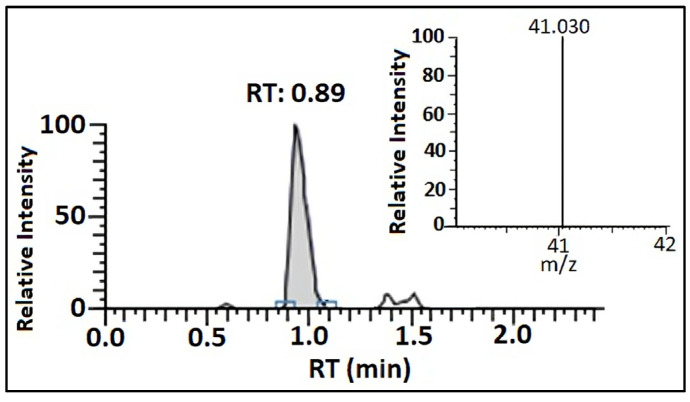
LC-MS/MS chromatogram of a blank maize sample spiked at LOQ level (5 μg/kg) and product ion spectrum of MON.

**Figure 2:**
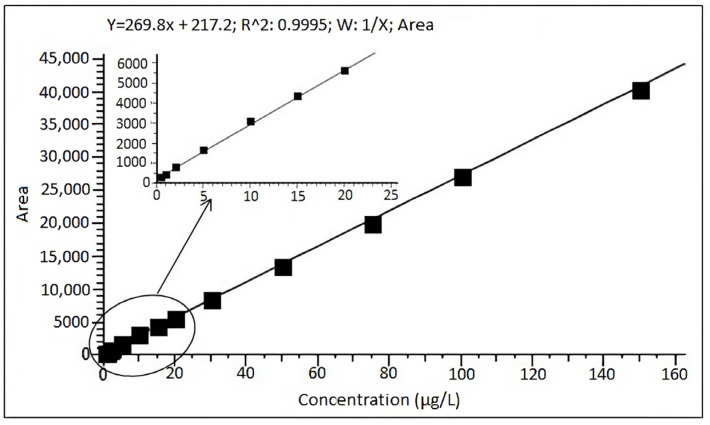
Calibration curve in the range from 0.5 to 150 µg/L of MON.

Using the proposed LC-MS/MS method, the following validation parameters were determined: LOQ, linearity, recovery, repeatability, reproducibility, and matrix effect. The LOQ value was determined at 5 μg/kg (Figure 1). The linearity was established in the range of 0.5–150 µg/L, which corresponded to 5–1500 μg/kg in the sample, with the squared correlation coefficient (R^2^) above 0.999 (Figure 2). The recovery was in the range of 87–103%, while reproducibility and repeatability are identified as relative standard deviations, with neither exceeding 20%. Furthermore, the matrix effect was in the range of 86–102%; therefore, MON was quantified using the solvent standard calibration curve (Figure 2).

Based on the obtained validation results, the applied LC-MS/MS method was considered suitable for the reliable determination of MON concentrations in unprocessed maize, and was in line with the criteria given in the Technical Report CEN/TR 16059 [25] and the European Regulation [26].

The method developed and validated in this study is the first LC-MS/MS method for MON determination in Serbia as well as in neighboring countries and therefore has great national as well as regional significance.

### 2.2. MON Occurrence in Different Production Regions and Years

The validated LC-MS/MS method was for the determination of natural MON occurrence in unprocessed maize samples in terms of the frequency, range, mean, and median levels of MON by region (Bačka, Banat, and Srem) and production year (2018–2021) (Table 1).

It can be observed from the results obtained that MON was detected and quantified in all 400 examined maize samples. The data suggest that the maize grown in the three primary maize growing regions (Bačka, Banat, and Srem) of Serbia over four years has a high risk of being contaminated with MON. Although the frequency of MON contamination was 100% in maize samples from each growing region, as well as from each year, differences between the concentration levels detected in the examined maize samples were observed.

From the analysis of samples by production year, it can be concluded that the highest MON concentration level was detected in 2021 (1742.0 µg/kg), while the highest concentration of MON-contaminated samples seen in the other three examined years was significantly lower (from 205.7 to 564.1 µg/kg). The mean concentrations varied from 41.2 µg/kg detected in 2019 to 222.7 µg/kg detected in 2021. Tukey’s post hoc test confirmed significant differences between mean concentrations of MON in samples from different years (Table 1). On the other hand, by comparing samples by production region in the same year with Tukey’s post hoc test, no significant differences could be confirmed between concentrations of MON in samples from different regions collected in the period 2018–2021, except for lower MON concentrations detected in maize samples from 2020 in the Banat region in comparison to the Bačka and Srem regions. These results were compared to other researchers’ results from Serbia and other countries, and are described in Section 2.4.

It seems that weather conditions in Serbia are favorable for the growth of *Fusarium* fungi and MON production. Therefore, this high frequency and these differences in concentration levels may be the results of weather condition parameters observed in the examined years. 

### 2.3. Impact of Weather Conditions

**Figure 3:**
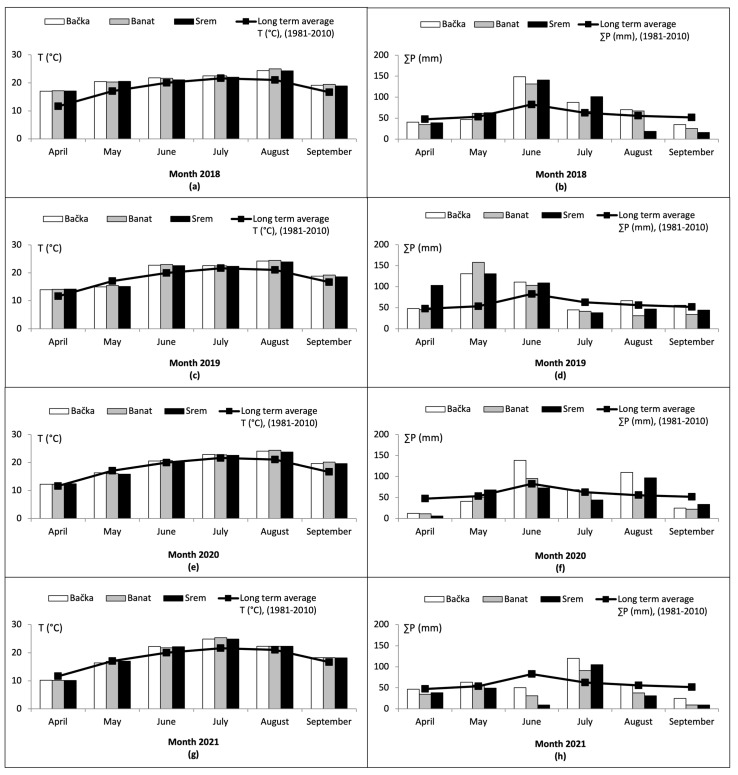
Monthly average air temperature and the sum of precipitation by growing region for the vegetation period (April−September) in 2018 (**a**,**b**), 2019 (**c**,**d**), 2020 (**e**,**f**), and 2021 (**g**,**h**) in comparison to long-term average values (1981−2010).

**Figure 4:**
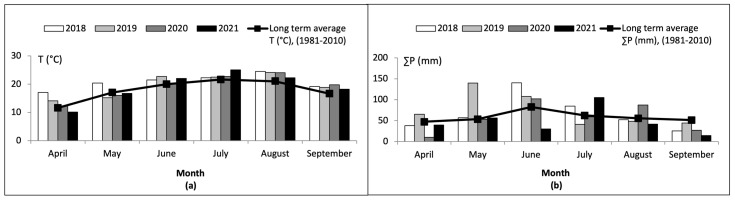
Monthly average air temperature (**a**) and sum of precipitation (**b**) in Northern Serbia by harvest year for the vegetation period (April−September) in the four-year period (2018–2021) in comparison to long-term average values (1981−2010).

The varying elements of the climate such as temperature, available water, extreme drought, quality and quantity of light, desertification, and humid or dry cycles are crucial factors that affect the life cycle of fungi in agroecosystems. Climate change can alter the ability of mycotoxigenic fungi to grow in certain areas and, consequently, produce toxins in crops. A shift in the geographical distribution and the pattern of mycotoxin occurrence is a possible outcome due to the ability of fungi to adapt to climate change [2,27,28]. Generally, the climate of Northern Serbia is moderate continental, with cold winters and hot and humid summers [29]. However, investigations in the past few years indicate that the impact of climate change has resulted in the Northern Serbian climate being marked by a wide range of extremely variable temperatures and irregular monthly precipitation patterns. Therefore, to elucidate the potential variations in the concentration levels of MON across different harvesting years and growing regions, we have compiled and presented the weather condition parameters including the average air temperature and the total amount of precipitation during the planting, growing, and harvesting season (April–September) from 2018 to 2021 in three regions of Northern Serbia (Bačka, Banat, and Srem), as illustrated in Figure 3 and Figure 4.

It can be noted (Figure 3a,c,e,g) that the monthly average air temperatures in the three examined regions in each study year were similar during the maize growing season, while the monthly average air temperatures in the three examined regions throughout the maize cultivation period were mostly higher than the average levels recorded over the long term (1981–2010). Only the monthly average air temperatures recorded during April 2021 and May 2019, 2020, and 2021 were found to be lower in all three regions than the average air temperatures recorded between 1981 and 2010. On the other hand, considerable differences were observed in the sum of precipitation between the three examined regions in each study year as well as compared to long-term average values (Figure 3b,d,f,h). In all three regions, May 2019, June 2018, and July 2021 experienced a high amount of rainfall, surpassing the long-term average values. Additionally, Srem had an extremely high amount of precipitation in April 2019, July 2018, and August 2020, while Bačka did in June and August 2020. Furthermore, in April 2020, June 2021, July 2019, and September 2018, 2020, and 2021, a significantly lower sum of precipitation occurred in all three regions. Additionally, Srem had a significantly lower sum of precipitation in July 2020 and August 2018 and 2021, while Banat did in August 2019 and 2021.

In terms of recorded weather conditions between the examined years, it can be noticed (Figure 4a) that the monthly average air temperatures during the maize growing season in each study year were mostly higher than the long-term average values (1981–2010). In comparison to the long-term average temperatures (1981–2010), the monthly average air temperatures recorded were lower only during April 2021 and May 2019, 2020, and 2021. On the other hand, significant variations are evident in the total rainfall recorded in the same timeframe in comparison to the established long-term average values (Figure 4b). Specifically, unusually high precipitation levels were registered in May 2019, June 2018, and July 2021, whereas April 2020, June 2021, and September 2018, 2020, and 2021 showed remarkably lower amounts of rainfall. The total amount of precipitation during the maize growing season (April–September) in 2021 was lower than the long-term average value, that in 2020 was around the long-term average value, and those in 2018 and 2019 were above the long-term average value (1981–2010).

In summary, recorded weather conditions during the growth period for maize, from April to September, across the four-year span from 2018 to 2021 can be described as follows: 2018 was warmer with slightly above-average rainfall; 2019 was warmer with a significantly greater amount of precipitation; 2020 was warmer with average humidity levels; and 2021 was warmer with a considerably lower amount of precipitation (Figure 3 and Figure 4). It can be seen that the monthly average air temperatures among the three examined regions and four examined years were similar during the maize growing season. On the other hand, there is a notable contrast observed in the sum of precipitation among the three examined regions and four examined years as well as compared to long-term average values. The mentioned weather conditions proved to be suitable for the synthesis of MON, given that the frequency and the total (100%) of MON was the same in samples from all three regions in the four examined years. Although the frequency of MON was 100%, and although there were no significant differences between concentrations of MON in samples from different regions in the same year, the differences between concentrations of MON in samples from different years were confirmed. The mean concentration in the year 2018 was rather similar to the 2019 and 2020 maize growing seasons, the mean concentrations in the years 2018 and 2019 were significantly different (*p* < 0.05), and the mean concentration in the year 2021 was significantly different (*p* < 0.05) and was higher than in the years 2018–2020. As already stated above, the highest mean (222.7 ± 215.4 µg/kg), median (154.0 µg/kg), and quantified concentration of MON (1742.0 µg/kg) were determined in 2021, whereas in 2018, 2019, and 2020, the mean concentration of MON was approximately 2 to 3 times lower. These differences could be due to the mentioned unusual changes in rainfall or temperature during the maize growing season. It can be noted that higher levels of MON were detected in maize from Serbia when higher temperatures and lower precipitation were observed in the growing season. This conclusion was also compared to other researchers’ results from Serbia and other countries, and is described in Section 2.4.

### 2.4. Comparative Study

The results from the available studies in the literature were investigated in an effort to provide a comparative understanding of the consequences of climate change for the presence of MON.

**Figure 5:**
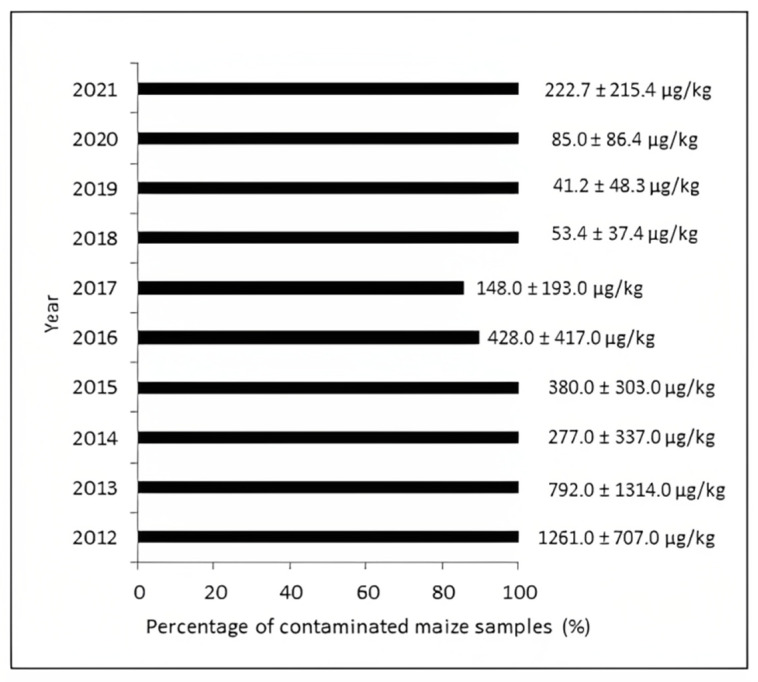
An overview of the percentage (%) and mean content (µg/kg) of contaminated maize samples with MON in Serbia during the ten-year period 2012–2021.

Until 2019, there were no data on the occurrence of MON in maize, nor in other matrices in Serbia; furthermore, in the period 2019–2023, only three articles were published about MON occurrence in Serbian cereals [6,8,30]. Due to the lack of LC-MS/MS, all three available investigations from Serbia were realized in Austria. Firstly, this comparative study was carried out to examine the presence of MON in maize collected from Serbia. Figure 5 presents an outline of the data for the various seasons during which maize was grown: 2012–2015 [8]; 2016–2017 [6]; and 2018–2021 (the results from the present study). The occurrence data are indicated in the form of percentage and mean concentration. As can be seen, an extremely high percentage of maize contamination with MON was observed; 86% in 2017, 90% in 2016, and 100% of maize samples collected from Serbia that were harvested in 2012–2015 and 2018–2021 were contaminated,. If we summarize the period of ten years (2012–2021), a total of 1062 maize samples were analyzed, 1008 maize samples contained MON, and the total frequency was 95%. Although a high frequency of MON was observed in the ten-year report, differences between concentration levels detected in examined maize samples were observed. The significant variations in MON concentration trends evident in maize samples gathered during various years can be attributed to climate change effects observed in Serbia over the ten-year assessment period. In addition, in all three compared studies, it was noted that higher levels of MON were detected when higher temperatures and lower precipitation were observed in the growing seasons. If the period of 10 years is summarized, the highest mean concentration of MON was detected in 2012 (1261.0 ± 707.0 µg/kg), and according to published data [8], the year 2012 was characterized by extreme drought.

Furthermore, Jajić et al. [30] confirmed a widespread occurrence of MON in maize grown in Serbia during a three-year period (2016–2018). In the year 2016, the level of contamination was the highest, and the presence of MON was found in 50% to 100% of the samples. The highest mean concentration was also detected in 2016 (920.1 µg/kg). It was confirmed that environmental conditions in Serbia facilitated the infection of maize ears by *Fusarium*, leading to a significant rise in MON contamination. Unfortunately, this study did not show MON contamination by year, but by clustering localities according to their administrative area into six distinct regions; therefore a valid comparison cannot be made with the results in Figure 5.

The results from this study, as well as previous studies from Serbia, are in agreement with previous studies on the widespread occurrence of MON worldwide. Many European countries have reported the presence of MON in cereals, with maize being the most frequently affected crop as per the available published data [14,23,24]. On the other hand, the fact that the level of maize contamination with MON is likely dependent on weather conditions during the maize growing season was highlighted by several authors. According to Scarpino et al. [31], between the years 2008 and 2011, 81–100% of the maize samples collected in Italy were found to be contaminated with MON, with the highest concentration level of 2606 µg/kg in 2008. The year 2008 was characterized by low growing degree days and high rainfall, from the early milk stage to the harvest. On the other hand, according to Bertuzzi et al. [19], 95.1% of maize samples collected in Italy in 2018 were found to be contaminated with MON, with the highest concentration of 4811 µg/kg. The researchers determined that in the absence of drought conditions, there was a strong and statistically significant link between growing degree days and MON values observed in the maize samples. A study from Germany [32] reported that maize samples were contaminated ranging from 43 to 45% during the harvesting years 2006 and 2007, with a maximum concentration of 3330 µg/kg detected in 2006. The year 2006 was characterized by high temperatures and low rainfall during anthesis and early grain filling. Based on this comparative study, it seems that the production of MON was favored to a great extent by temperature, while humidity depended from study to study. Concisely, in Italy, higher MON contamination levels were detected when warm and not droughty climate conditions were observed. In Serbia and Germany, the highest MON contamination levels were detected when high temperatures and low rainfall were observed. The reasons behind these variations may be attributed to the adaptive capacity of mycotoxigenic fungi to cope with environmental shifts caused by climate change. This adjustment can result in a repositioning of their range as well as a favorable climate for the growth of *Fusarium* species. According to certain findings, drier and warmer environments are associated with a higher prevalence of *F. proliferatum* and *F. verticillioides*, whereas *F. subglutinans* is frequently observed in cooler and more humid conditions [32]. Goertz et al. [32] investigated the occurrence of MON in German maize and found that in 2006, *F. graminearum*, *F. verticillioides*, and *F. proliferatum* were the primary species responsible for infecting maize kernels. However, in 2007, they found that the most commonly isolated species were *F. graminearum*, *F. subglutinans*, and *F. cerealis*. MON was detected as a common contaminant with similar frequency in both growing seasons, while the highest mean concentration was detected in 2006. A study conducted by Krnjaja et al. [33] investigated the natural occurrence of toxigenic fungal species in maize from Serbia. The identified species of *Fusarium* were *F. proliferatum*, *F. subglutinans*, *F. graminearum*, and *F. verticillioides*. Furthermore, according to the findings of Tančić Živanov et al. [34] in a recent study, 11 species of *Fusarium* were found in the grains of two different commercial maize varieties that were cultivated in Serbia. Among these species, *F. graminearum*, *F. verticilioides*, and *F. graminearum* were the most commonly identified. Therefore, it could be assumed that the same *Fusarium* species occurred in Serbian maize as in Germany, and MON was a very common contaminant in both studies. In addition, according to the profiles of *Fusarium* species, it seems that MON may be a common contaminant in maize regardless of the prevailing environmental conditions, while the concentration level depends on the observed weather conditions.

From the analysis of available studies related to the fact that the level of MON maize contamination likely depends on weather conditions, it is observed that only one study included maize samples harvested in the last ten years, but the study included maize samples from only one harvest year [19]. Therefore, according to the authors’ knowledge, this study represents the most extensive published study in Europe and beyond, and the authors believe that the results of this study contribute to increasing knowledge in the field of the presence of MON. Certainly, collecting additional data about its presence in maize grown in European countries may aid in a more comprehensive assessment of the agricultural and environmental factors that promote its growth. Based on everything analyzed above, additional investigation is needed to determine whether other agronomic elements, including prior crop, cultivation techniques, host variety, and fungicide usage, may have also impacted the incidence of *Fusarium* mycotoxins in the harvested cereals. There is also a need for MON reduction studies.

## 3. Conclusions

The present study describes the first developed and validated LC-MS/MS method in Serbia for the determination of MON in maize samples, as well as leading to a clearer comprehension of how weather conditions impact the frequency of MON in maize samples grown in fields of Northern Serbia. The detection of MON with an extremely high prevalence rate in maize crops in Serbia implies that the country could be susceptible to the presence of this mycotoxin. MON contamination in ten-year harvested maize showed year-to-year variations, with weather conditions as determinant factors. The findings suggest that dry and hot weather conditions resulted in the highest level of MON in maize samples. This study’s outcomes are seen by the authors as a substantial addition to our understanding of the occurrence of MON. To the best of the authors’ understanding, the present study constitutes the most extensive published study in Europe and beyond and the first report from Serbia that offers a comparative understanding of the prevalence of MON in maize samples obtained throughout a decade. However, high prevalence and co-occurrence with other regulated mycotoxins, the potential toxicity and toxicological synergistic effects with other mycotoxins, and rapidly increasing evidence of MON incidence show that regulatory bodies might need to increase surveillance in this area. We conclude by calling attention to the need for more research articles related to MON.

## 4. Materials and Methods

### 4.1. Sample Collection

A total of 400 unprocessed maize samples were collected in the time frame of four years from 2018 to 2021. To represent the annual production, 100 maize samples were collected every year after harvest (Table 2). The cultivation of samples and sampling were performed across three regions (Bačka, Banat, and Srem) in northern Serbia. The provisions of Serbian [35] and European Regulation [36] were adhered to while conducting sampling and preparing the samples. Briefly, 100 incremental samples were collected, composed of approximately 15 kg of aggregate maize samples. The aggregate samples were delivered to the Institute of Food Technology in Novi Sad. Homogenization of aggregate samples was performed using a Nauta mixer with a maximum capacity of 25 kg (Hosokawa Micron B.V., model 19387, Doetinchem, the Netherlands). Homogenized samples were quartered to obtain 1 kg laboratory sample and were further ground using a KnifetecTM mill (model 1095, Foss, Hoganas, Sweden). Before conducting the analysis, ground samples were stored in a freezer at a temperature of −20 °C. Later, in the year 2022, laboratory samples were retrieved from the freezer and processed through a homogenization process utilizing a 1 kg capacity Nauta mixer (Hosokawa Micron B.V., model 11102, Doetinchem, the Netherlands). The subsamples were then quartered from the homogenized material and used for further analysis.

### 4.2. Chemicals and Reagents

Acetonitrile (high-performance liquid chromatography (HPLC)-grade) purchased from Fisher Scientific (Geel, Belgium) and water purified using an Adrona Water Purification system (Riga, Latvia) were used for sample preparation. Water purchased from Fisher Scientific (Geel, Belgium), methanol purchased from Carlo Erba (Val de Reuil, France), and formic acid purchased from Fluka Analytical (Buchs, Switzerland), all of LC-MS/MS grade, were used for the analysis. MON standard at a concentration of 100.4 μg/mL was purchased from Romer Labs (Tulln, Austria). The working standard solutions were prepared in the concentration range from 0.5 to 150 µg/L by diluting the stock standard solutions in methanol:water (50:50, *v/v*). Correspondingly, standards that matched the matrix of the samples were created in the concentration range of 0.5 to 150 µg/L by adding a suitable volume of the standard stock solution to the blank sample extract and diluting it with a mixture of methanol and water in the ratio of 50:50 (*v/v*). The solutions of MON standard and stock were frozen and stored at −20 °C.

### 4.3. Sample Preparation

Sample preparation for LC-MS/MS analysis followed the protocol of Hofmann and Scheibner [37] with slight adaptations. In brief, a ground maize sample (5 g) was measured into a polypropylene tube of 50 mL capacity and extracted with acetonitrile:water (80:20, *v/v*). During the extraction, the samples were shaken using a horizontal shaker (Biosan, Latvia) at 350 rpm for 60 min and centrifugated (Boeco, Hamburg, Germany) at 4000 rpm for 5 min. A 400 μL aliquot of the filtered supernatant was diluted with 600 μL methanol:water (50:50, *v/v*) and filtered through a 0.2 µm polytetrafluoroethylene (PTFE) disposable syringe filter into vials.

### 4.4. LC-MS/MS Analysis

An HPLC Vanquish Core system comprising the Hypersil GOLD C18 Selectivity HPLC column, 100 × 2.1 mm i.d., 1.9 μm particle size, coupled to a TSQ Quantis Triple Quadrupole mass spectrometer and heated electrospray ionization (HESI) source (all from ThermoFisher Scientific, Waltham, MA, USA), was used for the detection and quantification. The analysis was carried out as described in the ThermoFisher Scientific Application Note 65969 [37], with several modifications. Briefly, the mobile phase consisted of water (95%) and methanol (5%), each with a 0.1% formic acid addition. The autosampler tray was adjusted to a temperature of 20 °C, and the column was set to 40 °C, with an injection volume of 10 μL. Due to the low molecular weight, only a single fragment ion could be generated from the precursor ion in this case [38,39]. Therefore, one characteristic product ion was monitored, which was used for quantification and as qualifiers (41.03) (Figure 1). The retention time of MON was 0.89 min.

Detection was performed in negative mode (2500 V), and the analyte was ionized through HESI and monitored in selected reaction monitoring (SRM) mode. Nitrogen was used as the sheath, auxiliary, and sweep gas. The gases were set according to the following: sheath 30 arbitrary units (Arb), auxiliary 6 Arb, and sweep 1 Arb. Argon gas with a pressure of 1.5 mTorr was utilized as the collision-induced dissociation (CID) gas. The temperature for the ion transfer tube and vaporizer were adjusted to 325 °C and 350 °C, respectively. The duration of each cycle was 0.5 s. The TSQ Quantis 3.2 Tune software and TraceFinder 5.1 software (ThermoFisher Scientific, Waltham, MA, USA) were used for system control, acquisition, and data processing.

### 4.5. Method Validation

The validation parameters were evaluated in accordance with the Technical Report CEN/TR 16059 [25] and the European Regulation [26]. Therefore, the performance of the applied LC-MS/MS method was evaluated in terms of linearity, recovery, repeatability, reproducibility, limit of quantification (LOQ), and matrix effect. The matrix effect was calculated as the signal suppression/enhancement ratio, i.e., the matrix-matched standard calibration and solvent standard calibration slope ratio. The LOQ refers to the lowest concentration that fulfills the validation criteria for recovery and repeatability. The recovery, repeatability, and reproducibility were evaluated by analyzing uncontaminated maize samples spiked at five concentration levels ranging from 5 to 500 µg/kg, in six replicates.

### 4.6. Weather Condition Analysis

A comprehensive investigation into the influence of weather conditions on the presence of MON in maize during its growing cycle was carried out by studying various weather parameters. The Republic Hydrometeorological Service of Serbia [40] provided weather-descriptive data on deviation from the average air temperature and a sum of precipitation. The data mentioned were gathered during the agricultural periods of maize cultivation, spanning from April 1st until September 30th, for four years, namely from 2018 until 2021. Variations were identified by contrasting these data against those compiled over an extended span of time (1981–2010).

### 4.7. Statistical Analysis

Differences in the mean content of MON between different regions for the same year and between different years were statistically evaluated using post hoc Tukey’s HSD test (*p* < 0.05). Statistical analysis was performed using Statistica software, version 14.0.0.15 (TIBCO Software Inc., Palo Alto, CA, USA).

## Figures and Tables

**Table 1:** The natural occurrence of MON in maize samples collected from the main maize production regions in Northern Serbia in four consecutive production years (2018–2021).

Year	Region	N ^1^	Min^2^	Max ^3^	Mean ± Std ^4^	Median ^5^
2018	Bačka	37 (100%)	7.1	134.5	49.8 ± 31.4 ^a^	40.5
	Banat	36 (100%)	7.1	121.3	50.5 ± 27.3 ^a^	44.5
	Srem	27 (100%)	6.9	205.7	62.1 ± 53.3 ^a^	50.0
	TOTAL	100 (100%)	6.9	205.7	53.4 ± 37.4 ^AB^	44.13
2019	Bačka	53 (100%)	5.9	400.3	46.1 ± 42.1 ^a^	31.8
	Banat	31 (100%)	6.2	98.5	34.4 ± 25.0 ^a^	30.0
	Srem	16 (100%)	7.3	182.2	38.3 ± 44.1 ^a^	23.9
	TOTAL	100 (100%)	5.9	400.3	41.2 ± 48.3 ^A^	30.7
2020	Bačka	55 (100%)	9.6	564.1	101.8 ± 107.2 ^b^	72.6
	Banat	38 (100%)	9.9	156.6	56.2 ± 39.2 ^a^	44.4
	Srem	7 (100%)	51.7	170.3	108.8 ± 42.8 ^ab^	113.9
	TOTAL	100 (100%)	9.6	564.1	85.0 ± 86.4 ^B^	61.9
2021	Bačka	50 (100%)	29.8	722.4	188.7 ± 142.3 ^a^	140.9
	Banat	37 (100%)	47.9	1742.0	246.5 ± 285.9 ^a^	154.6
	Srem	13 (100%)	30.5	603.5	285.5 ± 210.1 ^a^	209.5
	TOTAL	100 (100%)	29.8	1742.0	222.7 ± 215.4 ^C^	154.0

^1^ Number and percentage of contaminated maize samples; ^2^ minimum content (µg/kg); ^3^ maximum content (µg/kg); ^4^ mean content ± standard deviation (µg/kg); ^5^ median content (µg/kg). The presence of different letters in the same column indicates statistically significant differences (*p* < 0.05) between the values, as determined using the post hoc Tukey HSD test (a,b: differences between concentrations detected in different regions for the same year; A, B, C: differences between concentrations detected in different years).

**Table 2:** Number of collected maize samples by years and regions of Northern Serbia.

Region	2018	2019	2020	2021
Bačka	37	53	55	50
Banat	36	31	38	37
Srem	27	16	7	13
Total	100	100	100	100

## Data Availability

Data is contained within the article.
